# Prevalence and Determinants of Antibiotic Consumption in the Elderly during 2006–2017

**DOI:** 10.3390/ijerph17093243

**Published:** 2020-05-06

**Authors:** Silvia Portero de la Cruz, Jesús Cebrino

**Affiliations:** 1 Department of Nursing, Pharmacology and Physiotherapy, Faculty of Medicine and Nursing, University of Córdoba, Avda. Menéndez Pidal, S/N, 14071 Córdoba, Spain; 2 Department of Preventive Medicine and Public Health, Faculty of Medicine, University of Seville, Avda. Doctor Fedriani, S/N, 41009 Seville, Spain; jcebrino@us.es

**Keywords:** aged, anti-bacterial agents, public health, trends

## Abstract

Elderly people are a particularly important population with regard to antibiotic overuse, using around 50% more antibiotics per capita than younger adults. The aim of this study was to analyze the prevalence, associated factors and evolution over time of antibiotic consumption among the Spanish population aged ≥ 65 years from 2006 to 2017. A descriptive cross-sectional study was conducted using data from the Spanish National Health Survey in 2006, 2011/2012 and 2017, and from the European Health Survey in Spain in 2009 and 2014. The sample consisted of 26,891 non-institutionalized individuals ≥ 65 years. Antibiotic consumption was the dependent variable, and sociodemographic variables, lifestyle habits and health status were analyzed using a logistic regression model. The prevalence of antibiotic consumption was 4.94%, with a marked increase from 2006 (4.64%) to 2017 (5.81%) (*p* < 0.0001). Higher antibiotic consumption was associated with poor or very poor self-perceived health status, no polypharmacy and not having been in hospital during the previous twelve months, while a lower consumption was linked to being limited but not severely due to a health problem and not being at all limited.

## 1. Introduction

Antibiotics are essential drugs in the prevention or treatment of microbial infectious processes [1]. Nevertheless, the World Health Organization has reported that around 50% of antibiotic consumption is irrational, irresponsible or without any clinical need [2]. The elderly represents a particularly important population with respect to antibiotic overuse, using around 50% more antibiotics per capita than younger adults [3,4]. People aged ≥ 65 years currently constitute 19.40% of the Spanish population [5], and the Spanish National Statistics Office predicts that this proportion will have increased to 25.20% by 2033 [6]. Age-related physiological changes place the elderly at high risk for infectious diseases due to a combination of factors, including immune senescence, altered skin and mucosal barrier function, degenerative changes in bone and cartilage, as well as a reduction in respiratory capacity [7,8,9]. In addition, infectious diseases are a leading cause of hospital admission and death in this patient population [10].

Inappropriate antibiotic therapy in older people entails significant risks and potentially adverse consequences, including the risk of drug interactions, side effects related to age or disease-related changes in metabolisms, as well as risks associated with multidrug-resistant organisms and Clostridium difficile infections [9,11,12]. In fact, one of the biggest public health problems worldwide is the emergence of resistance among bacterial pathogens [13].

Between 2000 and 2015, the consumption rate of antibiotics increased 39% globally [14]. According to the European Surveillance of Antimicrobial Consumption Network, Spain is among the European countries with the highest rates, reaching 26 defined daily doses (DDD)/1000 inhabitants/day in 2018, which is higher than the mean 20.1 DDD/1000 inhabitants/day for the European Union during the same year [15].

In view of this situation, a number of studies have investigated the role of sociodemographic and clinical factors in the increasing antibiotic consumption around the world, so that prompt action aimed at encouraging appropriate antibiotic use can be taken [16,17,18,19,20]. Nevertheless, there are very few studies of antibiotic consumption in the Spanish population that focus on associated factors [21,22], especially among people aged ≥ 65 years old [23]. The aim of this study was to analyze the prevalence, associated factors and evolution over time of antibiotic consumption among the Spanish population aged ≥ 65 years from 2006 to 2017.

## 2. Materials and Methods

A cross-sectional study was carried out from December 2019 to February 2020 using the data obtained from the records of the Spanish National Health Survey (SNHS) 2006 [24], the European Health Survey in Spain (EHSS) in 2009 [25], the SNHS 2011/2012 [26], the EHSS 2014 [27] and the SNHS 2017 [28]. The SNHS and EHSS are representative surveys of the general population (representativeness is ensured by assigning a weighting coefficient to each participant) carried out by the Ministry of Health, Consumer Affairs and Social Welfare in partnership with the National Institute of Statistics. These surveys are conducted in non-institutionalised Spanish residents through personalized interviews. The sampling design was multistage probabilistic, stratified by census areas (first stage), family homes (second stage) and individuals (third stage). The inclusion criteria, for the data analyzed in the present study, were people aged ≥ 65 years old living in Spain.

The sample was representative of the older population residing in Spain and, originally, consisted of 33,306 subjects, but due to a lack of data for some of the variables studied, 6415 (19.26%) were excluded when the descriptive, bivariate and multivariate statistical analyses were carried out. For the current study, the total sample of participants numbered 26,891: 5322 in 2006; 4125 in 2009; 5244 in 2011/2012; 5862 in 2014; and 6338 in 2017.

The dependent variable was “antibiotics consumption”. This variable was assessed through the question “Have you taken antibiotics during the last two weeks?”. Participants who answered “yes” were considered consumers of antibiotics. The independent variables were: year of the surveys (2006, 2011/2012, 2009, 2014, 2017), gender (male, female), age (65–74 years, 75–84 years, ≥85 years), level of education (without studies, primary, secondary or professional training, university), marital status (single, married, widowed, separated/divorced), nationality (Spanish, foreigner), size of town of residence (<10,000 inhabitants, 10,000–100,000 inhabitants, >100,000 inhabitants). The variables related to lifestyle and health status used in the study were self-perceived health (very good, good, fair, poor, very poor), currently smoking (yes, no), consumption of alcoholic beverages in the two weeks prior to the survey (yes, no), hospitalization during the past twelve months (yes, no), degree of limitation due to a health problem for at least six months (severely limited, limited but not severely, not at all limited) and polypharmacy (yes, no). Polypharmacy was calculated using an identical question in all the questionnaires: “From the following list of types of medication, please tell me which one(s) you have consumed in the last two weeks?”. Subjects were classified as polypharmacy if they answered “yes” in five or more different medication groups (used to treat diseases such as colds, flu, throat or lung infections, symptoms such as pain or fever, or laxatives). Although no consensus has yet been reached on the number of medications that must be consumed to be considered polypharmacy, this figure (five medications) has been used in recent studies conducted in different countries [29,30,31]. The body mass index (BMI) was calculated from the self-reported body weight and height and categorized according to the World Health Organization [32] as: underweight (BMI < 18.50 kg/m^2^), normal-weight (BMI ranged between 18.50– and 24.99 kg/m^2^), overweight (BMI ranged between 25.00 and 29.99 kg/m^2^), and obesity (BMI ≥ 30 kg/m^2^). To identify subjects with an associated chronic condition, we used the self-reported affirmative answer to the presence of any of the following physician-diagnosed diseases (categorized as none, 1–2, ≥ 3): arterial hypertension, hypercholesterolemia, asthma, chronic bronchitis, diabetes and cancer.

The data obtained from these surveys are available in the National Institute of Statistics and Ministry of Health, Consumer Affairs and Social Welfare websites [24,25,26,27,28] in the form of anonymized microdata: no special authorizations are therefore required for their use. According to the SNHS and EHSS methodologies, the microdata files are anonymous and are available to the public. In accordance with Spanish legislation, when secondary data are used, there is no need for approval from an Ethics Committee. Data research is available as Supplementary File.

A descriptive analysis was performed by calculating the counts and percentages for the qualitative variables. The proportions of categorical variables were compared using the chi-square test for contingency tables or Fisher’s exact test if the number of expected frequencies was greater than 5. In addition, a logistic regression was performed to identify the variables associated with antibiotic consumption. All variables with a significant association in the bivariate analysis were included in the multivariate analysis. Raw and adjusted odds ratios were calculated with 95% confidence intervals. The Wald statistic was used to exclude one by one from the model any variables with a *p* ≥ 0.15 (backward methodical selection procedure). All the hypothesis contrasts were bilateral and in all the statistical tests, those with a 95% confidence level (*p* < 0.05) were considered significant values. The statistical power for all analyses conducted was 80%. The statistical analysis was carried out using IBM SPSS Statistics version 25 (IBM Corp, Armonk, NY, USA), licensed to the University of Córdoba and University of Seville.

## 3. Results

### 3.1. Sociodemographic, Lifestyle and Health Status Variables

Of the 42,157 subjects aged ≥ 65 years who were invited to participate in the SNHS 2006, EHSS 2009, SNHS 2011/2012, EHSS 2014 and SNHS 2017, 8851 individuals were excluded due to the following reasons: 383 refused to participate, 268 were absent from their homes and 23 were unable to participate in SNHS 2006; 1213 refused to participate, 825 were absent from their homes and 83 were unable to participate in EHSS 2009; 1488 refused to participate, 931 were absent from their homes and 71 were unable to participate in SNHS 2011/2012; 154 refused to participate, 40 were absent from their homes and 28 were unable to participate in EHSS 2014; 1841 refused to participate, 1435 were absent from their homes and 68 were unable to participate in SNHS 2017. 

Thus, the study was based on data from 33,306 individuals who accepted to participate. Of them, a total of 6415 (19.26%) not answered, at least, one variable, so that they also were excluded. In that sense, in SNHS 2006, 2520 individuals were excluded who did not answer to the following variables: antibiotic consumption (1.47%), level of education (0.84%), BMI (65.20%), marital status (0.39%), nationality (0.32%), polypharmacy (30.01%), chronic condition (1.05%) and consumption of alcoholic beverages (0.74%). In EHSS 2009, 1900 individuals were excluded who did not answer to the antibiotic consumption (0.31%), level of education (0.53%), BMI (40.43%), marital status (0.22%), polypharmacy (6.42%), chronic condition (0.22%), currently smoking (25.95%) and consumption of alcoholic beverages (25.92%). In SNHS 2011/2012, 652 individuals were excluded who did not answer to the antibiotic consumption (0.42%), BMI (86.35%), marital status (0.97%), polypharmacy (8.64%), chronic condition (0.70%), currently smoking (1.25%), consumption of alcoholic beverages (1.11%), hospitalization over the past twelve months (0.28%) and degree of limitation due to a health problem (0.28%). In EHSS 2014, 658 individuals were excluded who did not answer to the antibiotic consumption (1.17%), BMI (71.43%), marital status (0.70%), polypharmacy (21.66%), chronic condition (1.29%), currently smoking (1.64%), consumption of alcoholic beverages (1.87%) and degree of limitation due to a health problem (0.24%). In SNHS 2017, 685 individuals were excluded who did not answer to the antibiotic consumption (0.26%), BMI (83.72%), marital status (1.42%), polypharmacy (10.21%), chronic condition (0.90%), currently smoking (1.55%), consumption of alcoholic beverages (1.81%) and degree of limitation due to a health problem (0.13%).

Finally, the total sample was 26,891 records of older people, including 59% (*n* = 15,865) women and 41% *(n* = 11,026) men between 65 and 104 years old. The most frequent sociodemographic, lifestyle and health status characteristics of the participants were that they were married (51.90%), lived in towns with a population of over 100,000 inhabitants (41.70%), had no studies (44.67%), were Spanish (97.55%), had a fair self-perceived health status (35.03%), had no polypharmacy (78.53%), had, at least, one chronic condition (71.73%), were overweight (43.78%), were not current smokers (88.23%), had not consumed alcohol in the last two weeks (65.06%), had not been in hospital during the last twelve months (86.25%) and did not report any limitation due to a health problems in the last six months (57.50%).

Regarding the sociodemographic, lifestyle and health status variables of the sample according to the year of the survey (Table 1), a decrease in the number of older people without studies can be seen (2006: 82.71%, 2017: 29.38%; *p* < 0.0001). There was also a decrease in the number of participants who were overweight (2006: 46.37%, 2017: 44.38%; *p* < 0.0001).

### 3.2. Antibiotic Consumption

**Figure 1:**
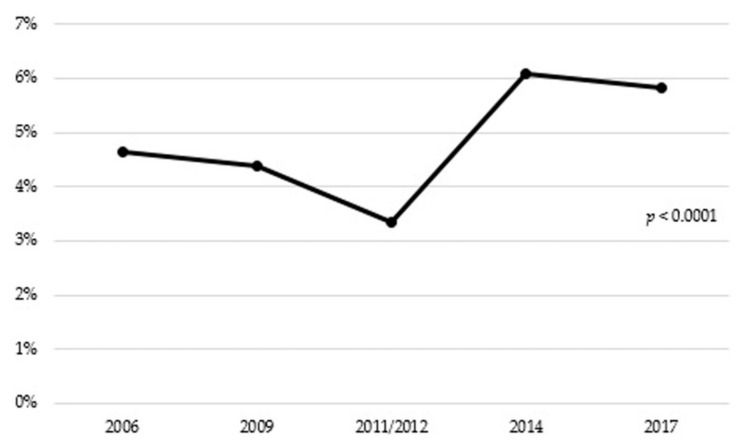
Antibiotic consumption in people aged 65 years or older in Spain (2006–2017).

The overall prevalence of antibiotic consumption was 4.94%. There were significant differences in antibiotic consumption between the different years of the study, with 4.64% consumption in 2006, compared to 5.81% in 2017 (*p* < 0.0001) (Figure 1).

### 3.3. Association between Sociodemographic, Lifestyle, Health Status Variables and Antibiotic Consumption.

The antibiotic consumption is distributed differently according to the sociodemographic, lifestyle and health status variables (Table 2). The bivariate analysis reveals a higher prevalence of antibiotic consumption in older people who reported not having polypharmacy (50.38%, *p* < 0.01) and currently being a non-smoker (90.81%, *p* < 0.01). A lower prevalence of antibiotic consumption was found in participants who had a very good self-perceived health status (3.69%, *p* < 0.0001), had no chronic diseases (16.42%, *p* < 0.0001), were severely limited (21.39%, *p* < 0.01) or had been in hospital in the last twelve months (29.74%, *p* < 0.01). In the multivariate analysis, from the year of 2014, the probability of antibiotic consumption in older people was higher than in 2006 and also in those had not been in hospital in the last twelve months. This probability was more than double in participants with no polypharmacy and a poor or very poor self-perceived health status. In contrast, a clear downward trend was observed in the probability of antibiotic consumption as the degree of limitation due to a health problem decreased. 

## 4. Discussion

The overall prevalence of antibiotic consumption in older people from 2006 to 2017 in Spain was 4.94%. This percentage was lower compared to previous studies conducted among the non-institutionalized elderly population, which ranged from 11% to 45%, with a significant rise over the past decade [23,33,34,35,36]. The variation observed may be due to multiple factors, rather than differences in health-care systems, including the number of general practitioners in a country, antibiotic dose regimens, guidelines, patients’ expectations and the attitude towards taking medication, cultural and social factors, as well as the source of information available to the general practitioners [37]. 

The trend of increases in antibiotic use from 2006 to 2017 among older adults shown in our study supports previous reports [23,38]. One factor that should be taken into account is the influence of seasonal fluctuation in the antibiotic consumption. A number of studies [39,40] have reported a remarkable seasonal variation in the antibiotic consumption in Spain with a winter predominance over summer, reflecting an inappropriate prescribing of antibiotic for respiratory tract infections caused by viruses. Nevertheless, in this study it was not possible to evaluate the seasonal fluctuation. The significant decrease in antibiotic use observed from 2009 (4.39%) to 2011/2012 (3.34%) may be due to the economic crisis that affected Spain from 2008 to 2013 [41], which led to the reduction of the purchasing power of elderly persons and the prescriptions were more closely controlled. Some authors have noted a decrease in the antibiotic consumption during that period [42]. After the economic crisis, in Spain, in 2014, there was an increase in the percentage of urinary and respiratory tract infections with respect to the period from 2009 to 2013 (16.7% and 11.1% increase, respectively) [43], which could explain the significant spike in antibiotic consumption observed in 2014. Likewise, in 2016, there was also an increase in the percentage of urinary and respiratory tract infections with respect to the period from 2011 to 2015 (22% and 5.5% increase, respectively) [44]. Although an increase in the percentage of infections is reported over the years, the antibiotic consumption decreased from 2014 to 2017. This situation may be explained because, in 2014, the Spanish Agency for Medication and Healthcare Pro-ducts promoted the creation of the National Programme against Antibiotic Resistance (PRAN) [45], in response to the European Commission request to address the problem following a common strategy [46]. In contrast, a constant rising trend of overall antibiotic use can be observed in national populations of older adults from Canada [47] and United States [48,49,50] and a decreasing trend in Denmark [51]. Whether this reflects changes in prescribing practices in this age group or differences in data sources is unclear.

Although women are about 27% more likely than men to receive antibiotic prescriptions due to the greater frequency of symptomatic infections of the urinary tract in women than in men, genetic and the over-use of primary healthcare services by women [52], this was not evident in the present study. Moreover, no relationship was found between antibiotic consumption and the participant’s age. While most studies point out that older people are more likely than younger adults to report having taken antibiotics [23,36,52], this increase can be explained by physiological changes in specific immune response patterns [52]. On the other hand, educational level was not associated with antibiotic consumption. In that sense, it has been reported the different role of education in developing countries, where it may improve access to medical treatment, than in developed countries, where access to medical treatment may not depend on educational levels [53,54]. Furthermore, in line with other studies [20,55], no relationship was found between antibiotic consumption and marital status. Regarding nationality, we found no significant differences between antibiotic consumption in Spanish and foreign elderly people. Cultural factors such as power distance, uncertainty avoidance and masculinity versus femininity are often referred to as possible explanations of differences in antibiotic consumption between people of different nationalities [56,57]. In addition, while the size of the town in which the participants lived was not associated with antibiotic consumption, city dwellers tend to report a higher use of antibiotics, which could be the result of having easier access to medical care, such as the greater availability of health care providers [58].

Despite the fact that we did not find any relationship between the BMI and antibiotic consumption, people who are overweight or obese are more likely to acquire an infection and thus need antibiotics compared with normal weight adults [59,60]. As found in another study [61], the likelihood of antibiotic consumption was greater in older people having a worse self-perceived health status, which may be because in a health system like the Spanish one, where access to care is universal, people with worse perceived health status visit their doctor regardless of their age. In the present study, the probability of antibiotic consumption was more than double in older people who had no polypharmacy. The concomitant use of multiple drugs can mask symptoms of infection, thus hampering diagnosis and necessitating prescription of broad-spectrum antimicrobial drugs to provide full antibiotic coverage [12,62]. As found by Palacios et al. [23], chronic diseases were significantly associated with the consumption of antibiotics. Patients with comorbidity and multimorbidity have increased susceptibility to infection due to poor mobility and/or perceptions around these patients’ risk of infection-related complications [63]. Regarding tobacco consumption, we found that antibiotic use was lower in non-smokers than in current smokers; similar results were found in another study [59]. Cigarette smoking compromises the immune system, leading to higher rates of respiratory and other infections [64,65]. Moreover, despite the fact that alcohol impacts both the innate and the acquired immune system and, thus, increases the risk of infectious diseases [66,67], in our study, no relationship was found between alcohol consumption and antibiotic use. 

Although hospitalization has been reported as a risk factor for antibiotic consumption [62], in our study, the probability of antibiotic consumption was higher in older people who had not been in hospital in the past twelve months. In fact, the vast majority of human consumption of antibiotics occurs in the community [15]. This may reflect the fact that patients need and demand quick relief from their symptoms, which seems to favor the prescription of antibiotics because doctors, especially those in the community, tend to respond to patients’ needs by prescribing antibiotics [68,69]. On the other hand, the probability of antibiotic consumption decreased as the degree of limitation was reduced. The results from a number of studies have suggested that a higher degree of limitation is related with increased risk of infection [12] and antibiotic resistance [70] in elderly persons. Furthermore, limitation due to a health problem is a predictor of mortality and a criterion for the early use of antibiotics in this population [12]. 

The results of this study should be taken into consideration by health authorities when designing (or improving current) guidelines and strategies for promoting the correct use of antibiotics, especially considering the elderly population. Improving antimicrobial stewardship is an international priority due to the evolving threat of antibiotic resistance. Continuous reassessments of the medication regime and current clinical status of older people by physicians and nurses are required to avoid the increase of antibiotic consumption and resistance.

The present study also has some limitations. First, due to the cross-sectional design, it is not possible to assign causality between the variables. Second, it should be remembered that the data collected in the survey was obtained indirectly from the informants’ self-reporting, which can be affected by memory and/or social desirability bias. Third, individuals can overestimate or underestimate their antibiotic consumption due to the self-reported measure of antibiotic consumption. Fourth, it was not possible to take into account the influence of seasonal factors in the antibiotic consumption and elderly people in institutions or hospitals have not been included. Fifth, since neither the SNHS not the EHSS identified specific active pharmaceutical ingredients (only groups of medicines for specific diseases or disorders) or the adequacy of antibiotic prescription, it would be advisable to include specific active pharmaceutical ingredients and a correct assessment of antibiotic prescription in future studies. Sixth, the amount of the total variance explained by the logistic regression model was modest, suggesting the importance of additional variables not explored by the different surveys. Seventh, BMI was calculated from height and weight reported by the participants, which may not be accurate. Lastly, although participation rate was relatively high, a non-participation bias cannot be ruled out, as it was not possible to compare the characteristics of the patients included in the study and those of who did not consent to participate. One strength of our study is that since the data were derived from a national survey, they have been obtained using carefully planned methodology, including sampling, well-designed forms, preparation of the survey participants, and supervision of the survey and filtering of the data, all of which guarantee a representative sample of the population.

## 5. Conclusions

In conclusion, the prevalence of antibiotic consumption in the Spanish population aged ≥ 65 years increased significantly from 2006 to 2017 and is still within the normal range of consumption. Nonetheless, it could potentially become a serious public health concern if the trend of antibiotic consumption continues to increase over time. The probability of antibiotic consumption is higher in older people with poor or very poor self-perceived health status, no polypharmacy and who had not been in hospital in the last twelve months, and is lower as the degree of limitation due to a health problem decreases.

## Figures and Tables

**Table 1:** Sociodemographic, lifestyle and health status variables of Spanish people aged ≥ 65 years (N = 26,891) (2006–2017).

Variables	2006	2009	2011/2012	2014	2017	*p*-Value
	*n* = 5322 (%)	*n* = 4125 (%)	*n* = 5244 (%)	*n* = 5862 (%)	*n* = 6338 (%)	
Gender						<0.0001
Male	2230 (41.90)	1655 (40.12)	1971 (37.59)	2469 (42.12)	2701 (42.62)	
Female	3092 (58.10)	2470 (59.88)	3273 (62.41)	3393 (57.88)	3637 (57.38)	
Age group (years)						
65–74	2855 (53.65)	2092 (50.72)	2433 (46.40)	2934 (50.05)	3181 (50.19)	
75–84	2048 (38.48)	1649 (39.98)	2070 (39.47)	2140 (36.51)	2268 (35.78)	<0.0001
≥85	419 (7.87)	384 (9.30)	741 (14.13)	788 (13.44)	889 (14.03)	
Level of education						<0.0001
Without studies	4402 (82.72)	1768 (42.86)	2080 (39.66)	1900 (32.41)	1862 (29.38)	
Primary	228 (4.28)	1505 (36.48)	1214 (23.15)	2542 (43.36)	2327 (36.72)	
Secondary or PT	546 (10.26)	584 (14.16)	1670 (31.85)	926 (15.80)	1546 (24.39)	
University	146 (2.74)	268 (6.50)	280 (5.34)	494 (8.43)	603 (9.51)	
Marital status						
Single	543 (10.20)	343 (8.32)	1570 (29.94)	497 (8.48)	505 (7.97)	
Married	2802 (52.65)	2141 (51.90)	2688 (51.26)	3005 (51.26)	3320 (52.38)	<0.0001
Widowed	1851 (34.78)	1526 (36.99)	608 (11.59)	2146 (36.61)	2218 (35.00)	
Separated/Divorced	126 (2.37)	115 (2.79)	378 (7.21)	214 (3.65)	295 (4.65)	
Nationality						<0.0001
Spanish	5265 (98.93)	4074 (98.76)	4858 (92.64)	5790 (98.77)	6244 (98.52)	
Foreigner	57 (1.07)	51 (1.24)	386 (7.36)	72 (1.23)	94 (1.48)	
Size of town of residence						<0.0001
<10,000 inhab	1712 (32.17)	1050 (25.45)	1522 (29.02)	1506 (25.69)	1640 (25.88)	
10,000–100,000 inhab	1638 (30.78)	1183 (28.68)	1550 (29.56)	1831 (31.24)	2045 (32.26)	
>100,000 inhab	1972 (37.05)	1892 (45.87)	2172 (41.42)	2525 (43.07)	2653 (41.86)	
Body Mass Index						<0.0001
Underweight	56 (1.05)	32 (0.78)	109 (2.08)	67 (1.14)	71 (1.12)	
Normal weight	1537 (28.88)	1173 (28.44)	2316 (44.16)	1816 (30.98)	1966 (31.02)	
Overweight	2468 (46.37)	1903 (46.13)	1965 (37.47)	2624 (44.76)	2813 (44.38)	
Obesity	1261 (23.69)	1017 (24.65)	854 (16.29)	1355 (23.12)	1488 (23.48)	
Self-perceived health						<0.0001
Very good	322 (6.05)	204 (4.95)	1140 (21.74)	389 (6.64)	399 (6.30)	
Good	1823 (34.25)	1501 (36.39)	2547 (48.57)	2231 (38.06)	2516 (39.70)	
Fair	2207 (41.47)	1653 (40.07)	1075 (20.50)	2129 (36.32)	2356 (37.17)	
Poor	750 (14.10)	597 (14.47)	403 (7.68)	828 (14.12)	836 (13.19)	
Very poor	220 (4.13)	170 (4.12)	79 (1.51)	285 (4.86)	231 (3.64)	
Polypharmacy						<0.0001
Yes	1048 (19.69)	1061 (25.72)	457 (8.71)	1515 (25.84)	1692 (26.70)	
No	4274 (80.31)	3064 (74.28)	4787 (91.29)	4347 (74.16)	4646 (73.30)	
Number of chronic diseases						<0.0001
None	1430 (26.87)	833 (20.19)	2774 (52.90)	1289 (21.99)	1274 (20.10)	
1–2	3295 (61.91)	2644 (64.10)	2112 (40.27)	3649 (62.25)	3768 (59.45)	
≥3	597 (11.22)	648 (15.71)	358 (6.83)	924 (15.76)	1296 (20.45)	
Currently smoking						<0.0001
Yes	455 (8.55)	322 (7.81)	1349 (25.72)	472 (8.05)	567 (8.95)	
No	4867 (91.45)	3803 (92.19)	3895 (74.28)	5390 (91.95)	5771 (91.05)	
Alcohol consumption						
Yes	508 (9.55)	1774 (43.01)	846 (16.13)	3010 (51.35)	3259 (51.42)	<0.0001
No	4814 (90.45)	2351 (56.99)	4398 (83.87)	2852 (48.65)	3079 (48.58)	
Hospitalization during the past twelve months						
Yes	774 (14.54)	683 (16.56)	468 (8.92)	841 (14.35)	931 (14.69)	<0.0001
No	4548 (85.46)	3442 (83.44)	4776 (91.08)	5021 (85.65)	5407 (85.31)	
Degree of limitation due to a health problem for at least six months						
Severely limited	491 (9.23)	416 (10.08)	184 (3.51)	728 (12.42)	635 (10.02)	<0.0001
Limited but not severely	1696 (31.86)	1730 (41.94)	894 (17.05)	2222 (37.91)	2434 (38.40)	
Not at all limited	3135 (58.91)	1979 (47.98)	4166 (79.44)	2912 (49.67)	3269 (51.58)	

PT: Professional Training; Inhab: Inhabitants.

**Table 2:** Association between antibiotic consumption and sociodemographic, lifestyle and health status variables in Spanish people aged ≥ 65 years (*n* = 26,891) (2006–2017).

Variables	Antibiotic Consumption				
	*n* = 1328				
	*n* (%)	OR (CI 95%)	*p*-Value	ORa (CI 95%)	*p*-Value
Year of the surveys					
2006	247 (18.60)	Reference		Reference	
2009	181 (13.63)	0.94 (0.77–1.15)	0.56	0.85 (0.69–1.04)	0.1
2011/2012	175 (13.18)	0.71 (0.58–1.86)	0.32	1.03 (0.84–1.27)	0.75
2014	357 (26.88)	1.33 (1.13–1.57)	<0.001	1.21 (1.02–1.44)	0.02
2017	368 (27.71)	1.27 (0.67–1.49)	0.08	1.17 (0.99–1.38)	0.07
Gender					
Male	511 (38.48)	Reference	0.06		
Female	817 (61.52)	1.12 (0.99–1.25)			
Age group (years)					
65–74	663 (49.92)	Reference			
75–84	502 (37.81)	1.00 (0.89–1.31)	0.94		
≥85	163 (12.27)	1.03 (0.87–1.23)	0.73		
Level of education					
Without studies	613 (46.16)	Reference			
Primary	365 (27.49)	1.21 (0.28–5.21)	0.8		
Secondary or PT	257 (19.35)	1.76 (0.14–4.02)	0.75		
University	93 (7.00)	3.43 (0.53–2.32)	0.2		
Marital status					
Single	124 (9.34)	Reference			
Married	713 (53.69)	0.91 (0.79–1.04)	0.17		
Widowed	436 (32.83)	0.95 (0.82–1.11)	0.53		
Separated/Divorced	55 (4.14)	1.02 (0.81–1.27)	0.87		
Nationality					
Spanish	1302 (98.04)	Reference	0.23		
Foreigner	26 (1.96)	1.27 (0.86–1.89)			
Size of town of residence					
<10,000 inhab	334 (25.15)	Reference			
10,000–100,000 inhab	431 (32.46)	1.17 (0.81–1.36)	0.08		
>100,000 inhab	563 (42.39)	1.12 (0.98–1.29)	0.1		
Body Mass Index					
Underweight	15 (1.13)	Reference			
Normal weight	409 (30.80)	1.04 (0.61–1.76)	0.89		
Overweight	551 (41.49)	1.05 (0.62–1.77)	0.86		
Obesity	353 (26.58)	1.34 (0.79–2.27)	0.28		
Self-perceived health					
Very good	49 (3.69)	Reference		Reference	
Good	296 (22.29)	1.41 (1.04–1.91)	0.03	1.25 (0.92–1.71)	0.15
Fair	506 (38.10)	2.79 (2.07–3.75)	<0.01	1.63 (1.19–2.23)	<0.01
Poor	360 (27.11)	5.79 (4.27–7.84)	<0.01	2.34 (1.67–3.27)	<0.01
Very poor	117 (8.81)	6.61 (4.67–9.32)	<0.01	2.14 (1.46–3.14)	<0.01
Polypharmacy					<0.01
Yes	659 (49.62)	Reference	<0.01	Reference	
No	669 (50.38)	3.94 (3.52–4.41)		2.57 (2.26–2.92)	
Number of chronic diseases					
None	218 (16.42)	Reference			
1–2	775 (58.36)	1.79 (1.53–2.08)	<0.01		
≥3	335 (25.22)	3.25 (2.73–3.88)	<0.01		
Currently smoking					
Yes	122 (9.19)	Reference	<0.01		
No	1206 (90.81)	0.75 (0.62–0.91)			
Alcohol consumption					
Yes	432 (32.53)	Reference	0.06		
No	896 (67.47)	0.89 (0.79–1)			
Hospitalization during the past twelve months					
Yes	395 (29.74)	Reference	<0.01	Reference	<0.01
No	933 (70.26)	2.85 (2.52–3.23)		1.89 (1.67–2.16)	
Degree of limitation due to a health problem for at least six months					
Severely limited	284 (21.39)	Reference		Reference	
Limited but not severely	586 (44.12)	0.53 (0.46–0.62)	<0.01	0.81 (0.69–0.96)	<0.01
Not at all limited	458 (34.49)	0.23 (0.20–0.27)	<0.01	0.67 (0.55–0.82)	<0.01

PT: Professional Training; Inhab: Inhabitants; OR: odds ratio; ORa: odds ratio adjusted for all sociodemographic, lifestyle and health status variables; CI 95%: 95% Confidence Interval; n: number of people consuming antibiotics. Nagelkerke’s R Square = 0.09.

## Supplementary Materials

The following are available online at https://www.mdpi.com/1660-4601/17/9/3243/s1, File S1: Research data.
